# Nanoparticles in the Diagnosis and Management of Stroke: A Review

**DOI:** 10.1155/srat/2132328

**Published:** 2026-10-09

**Authors:** Muhammad Furqan, Muhammad Qasim Qureshi, F. N. U. Huda, Hiba Azhar, Shaheera Younus, Saad Sohail, Saba Sattar, Sadia Binte Rahim, Muhammad Usman Haider, Mazhar Khalid, Bilal Butt, Tejas Phirke, Peter Kamel

**Affiliations:** ^1^ Department of Medicine King Edward Medical University Lahore Punjab Pakistan kemu.edu.pk; ^2^ Department of Neurology King Edward Medical University Lahore Punjab Pakistan kemu.edu.pk; ^3^ Department of Medicine University Medical and Dental College Faisalabad Punjab Pakistan duhs.edu.pk; ^4^ Department of Anesthesiology Jinnah Sindh Medical University Karachi Sindh Pakistan jsmu.edu.pk; ^5^ Department of General Surgery Jinnah Sindh Medical University Karachi Sindh Pakistan jsmu.edu.pk; ^6^ Department of Medicine Mymensingh Medical College Hospital Mymensingh Mymensingh Division Bangladesh mmc.gov.bd; ^7^ Department of Internal Medicine Geisinger Wyoming Valley Medical Center Wilkes-Barre Pennsylvania USA; ^8^ Department of Neurology Stony Brook University Stony Brook New York USA stonybrook.edu; ^9^ Department of Neurology University of Maryland School of Medicine Baltimore Maryland USA umd.edu; ^10^ Data Analytics Program School of Engineering and Applied Science George Washington University Washington DC USA gwu.edu; ^11^ Department of Neuroradiology Division of Diagnostic Imaging The University of Texas MD Anderson Cancer Center Houston Texas USA uth.edu

**Keywords:** blood–brain barrier, intracerebral hemorrhage, nanoparticle, neuroprotection, stroke, theranostics, translational nanomedicine

## Abstract

Stroke is the third leading cause of death and the fourth leading cause of disability‐adjusted life years worldwide, yet disease‐modifying treatment remains largely confined to reperfusion of large‐vessel ischemic occlusion within a narrow window. No neuroprotective or hemostatic agent has entered routine practice. Engineered nanoparticles have been proposed to improve delivery across the blood–brain barrier, concentrate drug within the ischemic or perihematomal microenvironment, and add imaging or sensing functions. The resulting literature is large but fragmented across platforms, models, and outcome measures, so it is not evident which platforms carry the strongest evidence or which are closest to clinical evaluation. This narrative review synthesizes preclinical and clinical evidence on nanoparticle‐based diagnosis, treatment, rehabilitation, and monitoring in ischemic and hemorrhagic stroke. We searched PubMed, Scopus, and the Cochrane Library from inception to July 2025 using predefined Boolean strings and assigned each included study an evidence tier to separate extrapolated and cross‐field evidence from stroke‐specific data. Comparative tables summarize nanoparticle type, payload, model, route, dose, treatment window, particle size and surface charge, principal outcomes, safety assessment, and translational readiness. Therapeutic evidence is almost entirely preclinical: Efficacy has been shown in young, male, comorbidity‐free rodents treated within minutes of reperfusion and followed for 24 h to 28 days, with group sizes typically between three and 15, and few studies reporting any bleeding or coagulation endpoint. Diagnostic applications are closer to patients because iron‐oxide nanoparticles have already been given to humans, although not for imaging. We identified seven registered clinical studies of nanoparticle‐related interventions in stroke; one perfluorocarbon nanodroplet emulsion completed a randomized dose‐escalation trial in acute ischemic stroke, the EU Clinical Trials Register returned none, and none exists in hemorrhagic stroke. Nanoparticle platforms, therefore, remain investigational, and progress will depend less on new chemistry than on comorbid and large‐animal models, realistic treatment windows, systematic reporting of bleeding risk and of interference with follow‐up imaging, and reproducible manufacturing.

## 1. Introduction

Stroke is defined as an episode of acute neurological dysfunction caused by focal infarction or hemorrhage of the brain, spinal cord, or retina, evidenced by imaging, pathology, or other objective findings, or by symptoms persisting at least 24 h when other causes are excluded [1]. It presents with abrupt focal deficit, most often unilateral weakness or sensory loss, facial droop, dysarthria or aphasia, visual field loss or ataxia, and, in hemorrhagic stroke, more often with headache, vomiting, and depressed consciousness [1, 2]. Diagnosis rests on the clinical syndrome with urgent neuroimaging, because computed tomography (CT) or magnetic resonance imaging (MRI) must separate ischemic from hemorrhagic stroke before any reperfusion treatment [2]. In 2021, there were an estimated 11.9 million incident strokes, 7.3 million deaths, and 160.5 million disability‐adjusted life years lost, making stroke the third commonest cause of death and the fourth commonest cause of disability‐adjusted life years worldwide [3]. Ischemic stroke, roughly four‐fifths of cases, follows arterial occlusion by thrombus or embolus and produces an irreversibly injured core surrounded by salvageable penumbra [2]. Hemorrhagic stroke comprises intracerebral hemorrhage, bleeding into the parenchyma, and aneurysmal subarachnoid hemorrhage, bleeding into the subarachnoid space, usually from a ruptured saccular aneurysm; the two differ in mechanism and management and are considered separately here [4, 5].

Treatment of ischemic stroke rests on restoring perfusion. Tissue plasminogen activator (tPA) (alteplase) is a recombinant serine protease that converts fibrin‐bound plasminogen to plasmin and so lyses the occluding thrombus; it is given intravenously within 4.5 h of onset, and tenecteplase, a variant with greater fibrin specificity and a longer half‐life, is an accepted alternative [2, 6, 7]. Mechanical thrombectomy is endovascular retrieval of the clot through a catheter, and pooled individual‐patient data from five randomized trials reduced 90‐day disability, with an adjusted common odds ratio of 2.49 (95% CI 1.76–3.53) and a number needed to treat of 2.6 [8]. Both are limited by narrow windows, restrictive eligibility, and reperfusion injury [6, 7]. Despite more than a thousand experimental treatments tested in animals, no neuroprotective agent has reached routine use, a gap attributed as much to methodological weakness in preclinical work as to species differences [9, 10]. Delivery is a further constraint, because the blood–brain barrier excludes most systemically administered agents from the parenchyma [11]. Advances in hemorrhagic stroke are scarcer: Guidelines recommend no disease‐modifying pharmacotherapy for secondary injury after intracerebral hemorrhage, and oral nimodipine remains the only agent recommended after aneurysmal subarachnoid hemorrhage [4, 5].

Nanomedicine applies engineered materials with at least one nanoscale dimension to diagnosis, monitoring, and treatment. Nanoparticles (NPs) are its principal vehicles, conventionally defined as particulate structures with external dimensions between approximately 1 and 100 nm, although carriers used in medicine commonly extend to several hundred nanometers; the constructs discussed here range from roughly 3 nm inorganic nanodots to particles above 300 nm [12, 13]. Their appeal in stroke lies in properties that free drugs lack: a high surface area available for functionalization, protection of labile payloads such as recombinant proteins and nucleic acids, tunable circulation time, and release triggered only by conditions present in the ischemic or perihematomal microenvironment [12]. The literature is nevertheless fragmented. Studies differ in chemistry, model, route, dose, and treatment window; head‐to‐head comparisons are rare, and the fraction of an administered dose reaching target tissue is small and inconsistently reported [14]. Toxicity, biodistribution, manufacturing reproducibility, regulation, and patient heterogeneity are frequently acknowledged but seldom measured [15, 16].

The gap this review addresses is, therefore, not a shortage of candidate NPs but the absence of a synthesis placing them on common evidence footing. Existing reviews are organized by material class and tend to report positive findings without recording the model, treatment window, safety package, or level of evidence behind each result, which makes it difficult to judge which platforms are genuinely near clinical evaluation. This narrative review, therefore, is aimed at (i) summarizing the principal NP platforms and targeting strategies used in stroke, restricted so far as possible to stroke‐specific evidence and with cross‐field examples labeled as extrapolated; (ii) appraising diagnostic and therapeutic applications against the decisions actually made in acute stroke care; (iii) addressing ischemic and hemorrhagic stroke separately; (iv) tabulating the comparative evidence for each platform, including model, species, route, dose, treatment window, particle size and surface charge, outcomes, toxicity assessment, follow‐up, and translational readiness; (v) identifying every registered clinical study of a NP‐related intervention in stroke; and (vi) setting out the barriers, including bleeding risk, interference with follow‐up imaging, manufacturing, and regulation, that separate current preclinical results from clinical evaluation.

## 2. Methods

This is a narrative review. It was not registered, and it does not claim the duplicate screening, risk‐of‐bias appraisal, or quantitative synthesis required of a systematic or scoping review. The search strategy is reported in full so that readers can judge and reproduce the evidence base. Still, no PRISMA flow diagram is presented because screening was not conducted with the stage‐by‐stage record‐keeping that such a diagram represents.

We searched PubMed, Scopus, and the Cochrane Library from inception to July 31, 2025, with the final searches executed in July 2025. The PubMed strategy combined three concept blocks with the Boolean operator AND, with keywords including “Stroke,” “Brain Ischemia,” “Intracranial Hemorrhages,” “Nanoparticles,” “Nanomedicine,” “Nanostructures,” “Blood‐Brain Barrier,” “neuroprotection,” “thrombolysis,” “diagnosis,” “imaging,” and “rehabilitation.” Equivalent TITLE‐ABS‐KEY strings were run in Scopus, and the population and intervention blocks were run in the Cochrane Library to capture controlled trials. We hand‐searched the reference lists of included articles and recent reviews.

Records were limited to full‐text articles in English. Studies were eligible if they reported original data on the diagnostic, therapeutic, rehabilitative, or monitoring use of a nanoscale material in a stroke‐relevant system, defined as an in vitro model of cerebral ischemia or hemorrhage, an animal model of ischemic or hemorrhagic stroke, or human participants. We retained reviews and papers from adjacent fields only for mechanistic or contextual background and identified them as such. Conference abstracts, editorials, and studies of nanomaterials with no stated stroke application were excluded. The review team removed duplicates before screening and assessed titles, abstracts, and full texts against these criteria, resolving disagreements through discussion.

Because a narrative review necessarily mixes evidence of different strength, each cited study was assigned an evidence tier, stated wherever a finding is described: in vitro (cell‐free or cell culture systems, e.g., oxygen–glucose deprivation and clot lysis assays), small‐animal in vivo, large‐animal in vivo, and human. Secondary reviews are cited for background or synthesis only and are never treated as equivalent to primary data. Crossfield evidence, such as work in glioma or perinatal brain injury, is labeled explicitly as extrapolated. Chemical formulae, particle dimensions, doses, and quantitative outcomes were verified against the primary publications rather than intermediate secondary sources. Sizes are hydrodynamic diameters by dynamic light scattering unless stated otherwise; where a source reports an outcome only graphically, its direction and significance are described, and no numerical value is quoted; and a treatment window is the interval between the onset of the experimental insult and the first dose.

## 3. Types of NPs

NPs used in medicine are grouped by the composition of their core, each class offering a different balance of biocompatibility, payload capacity, stability, and functionalization chemistry (Figure 1) [12, 13]. The classes below are those for which stroke‐relevant data exist, and the emphasis in each subsection is on what has been shown in a model of cerebral ischemia or hemorrhage rather than on the general properties of the material.

**Figure 1:**
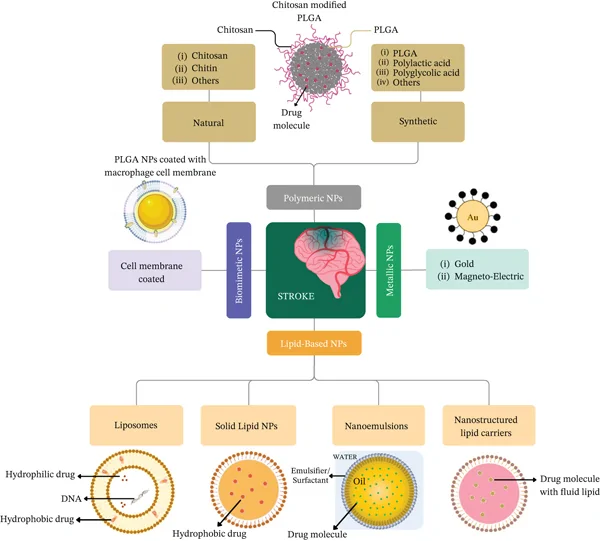
Classification of nanoparticle platforms used in stroke diagnosis and therapy.

### 3.1. Lipid‐Based NPs

Lipid‐based systems comprise liposomes, solid lipid nanoparticles (SLNs) and nanostructured lipid carriers (NLCs). Liposomes are vesicles formed by one or more phospholipid bilayers enclosing an aqueous core, so hydrophilic drugs are carried in the core and lipophilic drugs within the bilayer [17]. SLNs are submicron particles built from a lipid matrix that is solid at body temperature, giving physical stability but limited loading, because the crystalline matrix expels drug during storage [18]. NLCs blend a liquid lipid into that matrix, creating an imperfect lattice with more room for drug and less expulsion on aging [19]. All three are biocompatible because their constituents resemble endogenous membrane lipids, and their surfaces can be functionalized with antibodies or peptides to bind the inflamed endothelium of the ischemic and perihematomal vasculature [20]. Early liposomes were cleared within minutes: On contact with plasma, they adsorb opsonins, principally immunoglobulins, complement components, and fibronectin, and the resulting protein corona is recognized by the mononuclear phagocyte system, so particles are taken up by hepatic Kupffer cells and splenic macrophages [21]. PEGylation, the covalent attachment of polyethylene glycol, creates a hydrated steric barrier that reduces opsonin adsorption and prolongs circulation [21]. In stroke models, liposomes have delivered the iron chelator deferoxamine to limit reperfusion injury and atorvastatin to produce neuroprotection [22, 23].

### 3.2. Polymeric NPs

Polymeric NPs offer biocompatibility, physical stability, and control over release kinetics, because the drug is released as the polymer matrix hydrolyses and the rate can be tuned through polymer composition and molecular weight [24]. Poly(lactic‐co‐glycolic acid) (PLGA) is the most widely used example and is approved for parenteral use; coating PLGA NPs with erythrocyte membranes prolongs circulation and improves efficacy in thrombosis models [24, 25].

### 3.3. Metallic and Biomimetic NPs

Metallic NPs, of which gold nanoparticles (GNPs) are the most studied, are versatile carriers whose surfaces are readily functionalized through thiol chemistry. Their safety is not an intrinsic property of the material: Toxicity depends on core size, shape, surface coating, dose, route, biodistribution, and clearance, and small cationic particles have shown size‐dependent cytotoxicity [15, 26]. The most relevant stroke evidence is a small‐animal in vivo study in which apolipoprotein E‐mimetic peptide‐coated GNPs (38.12 ± 1.7 nm) given intranasally to rats 90 min after middle cerebral artery occlusion reduced infarct size from 21.2*%* ± 3.9*%* to 10.8*%* ± 4.6*%*; a 7‐day repeated‐dose study at 7 *μ*g/kg/day showed no change in serum biochemistry and no histological abnormality, but the authors state that the safety of longer or higher dose administration remains undetermined [27]. Metal‐organic framework (MOF) NPs are crystalline coordination polymers in which metal ions or clusters are bridged by organic linkers into a porous lattice of very high surface area, allowing exceptional drug loading [28]. MIL‐101(Cr), built from chromium(III) trimers and terephthalate linkers, has mesoporous cages among the largest reported for a crystalline solid, and PEGylation renders it colloidally stable in biological media; in vitro, PEGylated MIL‐101(Cr) reduced reactive oxygen species (ROS) accumulation and improved survival in SH‐SY5Y cells subjected to oxygen–glucose deprivation and was taken up by rat brain capillary endothelial cells, which suggests but does not demonstrate blood–brain barrier transit [29]. Magnetoelectric nanoparticles (MENPs) are core–shell composites, typically a magnetostrictive core within a piezoelectric shell, in which an applied magnetic field deforms the core and the resulting strain generates a local electric field, so an external magnet can stimulate electrically without an implanted electrode. In mice, intravenously administered MENPs were magnetically concentrated in the cortex and, at fields of 350–450 Oe, evoked reproducible neuronal calcium responses without genetic modification or detectable neuroinflammation [30]. That work was performed in healthy animals rather than a stroke model and is included as extrapolated evidence. Biomimetic NPs address rapid immune clearance by coating a synthetic core with membranes from erythrocytes, platelets, leukocytes, stem cells, or tumor cells, so the particle retains the surface proteins of the source cell, including the CD47 signal that suppresses phagocytosis, and inherits its tropism, such as a macrophage membrane′s homing to inflamed tissue [31, 32]. In a mouse ischemic stroke model, neural stem cell membrane nanovesicles crossed the barrier through a VCAM‐1/VLA‐4 interaction, and blocking VCAM‐1 reduced their entry by approximately 60% [32].

## 4. Targeting Strategies for NP Delivery

### 4.1. Passive Targeting

NPs can accumulate at sites of injury without a targeting ligand. In tumors and, by extension, in the ischemic core and its margin, defective microvasculature combined with impaired lymphatic drainage allows particles above the renal filtration threshold to extravasate and be retained; this is the enhanced permeation and retention (EPR) effect [33]. Size, shape, surface charge, and deformability determine circulation half‐life, margination toward the vessel wall, and organ distribution, and, therefore, how much passive accumulation is achievable: Particles below approximately 6 nm are cleared renally, particles above roughly 200 nm are captured by spleen and liver, and strongly cationic surfaces accelerate opsonization [13]. Two qualifications matter. The EPR effect was characterized in tumors, and its magnitude, consistency, and mechanism have been challenged, with the median delivered fraction of an injected dose well below 1% [14, 33]. In stroke, blood–brain barrier permeability is not fixed but changes over hours to days after the insult, so the window for passive accumulation is variable [11, 34].

### 4.2. Active Targeting

Active targeting conjugates ligands, aptamers, peptides, or antibodies to the particle surface so they engage receptors overexpressed on the target cell or the endothelium serving it, which increases the fraction of accumulated particles that are internalized and can reduce off‐target exposure; it depends on a difference in receptor expression large enough to exploit and does not by itself increase the total quantity delivered to the organ [35]. Stroke‐specific examples include AMD3100 engaging CXCR4 upregulated on ischemic brain endothelium [36], cyclic arginine–glycine–aspartic acid peptides directed at the activated‐platelet integrin *α*IIb*β*3 [37], fibrin‐avid peptides in thrombolytic constructs [38], and RVG29, a 29‐amino‐acid peptide derived from the rabies virus glycoprotein that binds the nicotinic acetylcholine receptor at the barrier [39]. Cytokine‐directed targeting has been explored, although the available demonstration is from oncology and is included as extrapolated evidence: Interleukin‐13‐conjugated liposomes bound IL‐13R*α*2‐overexpressing glioma cells and increased intracellular doxorubicin retention [40]. That study used a subcutaneous rather than orthotopic xenograft, so it demonstrates receptor‐mediated targeting but not blood–brain barrier transit and provides no evidence in cerebral ischemia.

### 4.3. Stimuli‐Responsive Drug Release

Stimuli‐responsive systems release payload only on encountering a trigger present in the target microenvironment. The ischemic brain provides several triggers: acidosis, hypoxia, elevated ROS, and increased protease activity, including the matrix metalloproteinases [41]. In a small‐animal in vivo study, Mei et al. developed a pH‐responsive polyion complex micelle carrying hirudin within an antioxidant nitroxide‐bearing polymer (HD@iNanoAOX) (65.2 ± 3.0 nm, zeta potential −7.8 ± 0.5 mV). Encapsulation extended the circulating half‐life of hirudin from approximately 21.9 to 91.5 min, and a single intravenous dose of 1.0 mg/kg hirudin 10 min after occlusion significantly reduced infarct volume, brain superoxide and malondialdehyde, Evans blue extravasation, and brain water content relative to saline, free hirudin, and a carrier lacking the antioxidant moiety [42]. Two caveats apply: Free hirudin tended to increase subarachnoid hemorrhage area, whereas the encapsulated formulations did not, which is the more clinically interesting finding, and although the authors describe the results as ischemia–reperfusion, the model was permanent photothrombotic occlusion with no reperfusion step, with all outcomes measured at 24 h in groups of five.

Sensitivity to pH is commonly conferred by incorporating phosphatidylethanolamine, which adopts a nonbilayer hexagonal phase when its headgroup is protonated, together with a weakly acidic stabilizer such as oleic acid, so the vesicle destabilizes in an acidic compartment [43]. Hydrogen peroxide‐responsive NPs have delivered urokinase, exploiting the high ROS concentrations at sites of thrombosis and inflammation [44]. Matrix metalloproteinases are upregulated in acute stroke and contribute to neurovascular injury [41], and peptide linkers cleaved by these enzymes permit site‐specific release, although the available demonstrations are largely from oncology and are cited as extrapolated evidence.

## 5. NPs in Stroke Diagnosis

Acute stroke imaging serves a small number of specific decisions: excluding hemorrhage before thrombolysis, identifying large‐vessel occlusion treatable by thrombectomy, estimating irreversibly injured core against salvageable penumbra, and predicting hemorrhagic transformation [2, 45]. An NP‐based diagnostic must improve one of those decisions within the minutes available to be clinically meaningful. Table 1 compares the agents on the parameters that determine whether they could be used in that pathway.

**Table 1:** Nanoparticle‐based imaging agents in stroke, compared with their clinical comparators. NR, not reported.

Modality	Agent	Model	Versus clinical comparator	Half‐life and clearance	Status
MRI, 3 T	BSA‐MnO2 (BM NPs) [46]	3 h tMCAO, rats	r1 5.9 vs. 4.25 mM^−1^ s^−1^ for Gd‐DTPA; predicts hemorrhagic transformation, AUC 0.867, 90% sensitivity	NR; organ manganese normal by Day 7	Preclinical
MRI, 7 T, and photoacoustic	MnCO3@BSA‐ICG [47]	90 min tMCAO, mice	r1 7.03 mM^−1^ s^−1^ at pH 6.5, field strength not stated; signal to 5 h	NR	Preclinical
CT, clinical scanner	PEGylated BaHoF5 [48]	4 h permanent MCAO, rats	~2x iohexol; artery 38.5–105 vs. 15 HU maximum for iohexol, which missed the infarct	88 min in mice; hepatobiliary	Preclinical
MRI, 7 T, T2∗	Platelet membrane magnetic carriers [49]	Photothrombotic, mice	No relaxivity or comparator reported; texture entropy separates microvascular from large‐vessel injury	NR	Preclinical
Magnetic particle imaging	SPIO tracers [50]	tMCAO, mice, *n* = 3	Cerebral blood flow drops 89.2% vs. 91.5% by MRI; resolution 330 *μ*m to 2 mm	Tracer‐dependent; reticuloendothelial	No human imaging performed; no approved MPI tracer
Optical biosensor	Upconversion nanoparticles, anti‐GFAP [51]	Human serum, spiked	Linear response 5–5000 pg/mL GFAP	Not applicable	Analytical proof of concept
Fiber‐optic gold immunoassay	FONLISA [52]	NT‐proBNP; buffer, serum, 13 plasma samples	Linear range 0.50–5000 pg/mL; limit of detection 0.058 pg/mL in buffer	Not applicable	Research assay
Clinical MRI, off‐label	Ferumoxytol [53]	Human	Macrophage uptake gives delayed enhancement	Reticuloendothelial	Approved for iron deficiency anemia only; imaging use is off‐label

Abbreviations: AUC, area under the receiver operating characteristic curve; GFAP, glial fibrillary acidic protein; HU, Hounsfield units; NT‐proBNP, N‐terminal pro‐B‐type natriuretic peptide; SPIO, superparamagnetic iron oxide.

### 5.1. CT

CT is the cornerstone of initial evaluation. Noncontrast CT differentiates hemorrhagic from ischemic stroke because acute blood is hyperdense [45], but it has low sensitivity for ischemic change in the first hours, so CT angiography and CT perfusion are commonly added [2, 54].

Researchers have investigated NPs as CT contrast agents because high‐atomic‐number elements attenuate X‐rays more strongly than iodine at diagnostic energies and because particulate agents circulate longer than small, iodinated molecules. In a small‐animal in vivo study, PEGylated BaHoF_5_ NPs (7 nm core, 30 nm hydrodynamic diameter) gave approximately twice the attenuation of iohexol at equal concentration; in rats imaged 4 h after permanent middle cerebral artery occlusion, arterial enhancement rose from 38.5 to 105 Hounsfield units within about 6 s, and perfusion maps showed the ischemic territory, whereas iohexol at the same dose produced a maximum enhancement of 15 Hounsfield units and failed to demonstrate the infarct [48]. The blood terminal half‐life of these particles was 88 min in mice, with predominantly hepatobiliary elimination, which the authors advance as an advantage in patients at risk of contrast‐induced nephropathy [48]. Longer circulation is achievable with surface engineering: 18 nm GNPs coated with 2 kDa polyethylene glycol showed a blood half‐life of approximately 4 h, rising to approximately 51 h with 10 kDa polyethylene glycol, against less than 10 min for a clinically used iodinated agent [55]. None of these agents has entered human use.

### 5.2. MRI

MRI is highly sensitive for ischemic brain injury. Diffusion‐weighted imaging detects ischemic lesions as hyperintense areas within hours of onset, and MRI visualizes small lesions in the posterior fossa and brainstem that bone artifact obscures on CT [56].

Gadolinium chelates are small molecules that distribute into the extracellular space, are cleared renally within minutes, cannot be targeted, and have been associated with nephrogenic systemic fibrosis and with gadolinium retention in brain and bone [54]. Superparamagnetic iron oxide (SPIO) NPs were developed as an alternative, being retained intravascularly for longer and amenable to surface functionalization [54]. Ferumoxytol is the only iron oxide NP in routine human use, and its status should be stated precisely: It is approved solely for the treatment of iron deficiency anemia, all imaging use is off‐label, and it carries a boxed warning for serious hypersensitivity [53]. Blood–brain barrier permeability itself carries prognostic information, because the degree of early disruption predicts hemorrhagic transformation, and it can be quantified by dynamic contrast‐enhanced imaging [34]. PEGylated SPIO NPs have been used to image barrier permeability and ischemic lesions concurrently on T2‐weighted MRI [54].

Bovine serum albumin–templated MnO_2_ nanoparticles (BM NPs) (8.9 nm, −20.0 mV) were used as a manganese‐based alternative to gadolinium for barrier permeability imaging in rats subjected to 3 h of middle cerebral artery occlusion. Longitudinal relaxivity was 5.9 mM^−1^ s^−1^ at 0.5 T against 4.25 mM^−1^ s^−1^ for Gd‐DTPA under identical conditions. Enhancement appeared within 5 min; in animals that developed hemorrhagic transformation, the peak signal was twice that of the contralateral hemisphere and persisted for almost 3 h, whereas in those without it, the signal was faint and had faded by 1 h. Combining peak intensity, duration, and extent predicted hemorrhagic transformation with an area under the curve of 0.867, and enhancement beyond 2 h gave 90% sensitivity and 70% specificity; a 14‐day toxicity study showed no change in body weight, 16 serum biochemical indices, organ histology, or open‐field behavior [46]. A manganese carbonate construct, MnCO_3_@BSA‐ICG (approximately 100 nm), is a dual‐modal magnetic resonance and photoacoustic agent with a pH‐dependent relaxivity of 7.03 mM^−1^ s^−1^ at pH 6.5; in mice after 90 min of occlusion, magnetic resonance signal rose within 30 min and persisted at least 5 h, photoacoustic signal was detectable from 1 h, and oxygen saturation in the ischemic hemisphere was 13.19% against 41.64% in normal brain [47]. Platelet membrane biomimetic magnetic nanocarriers (PAMNs) (218.43 ± 2.80 nm) delineated the vascular injury network in mice within 30 min, and the texture entropy of the T2∗‐weighted signal distinguished diffuse microvascular injury from large‐vessel occlusion, in which the particles could not reach the infarct core [49]. All three remain preclinical, none has been compared against a clinical agent at an equivalent dose in the same animal, and none reports a circulating half‐life.

### 5.3. Emerging Imaging Modalities

Magnetic particle imaging (MPI) is a tracer‐based tomographic modality that detects the nonlinear magnetization response of SPIO NPs to an applied field; because it detects the tracer directly rather than its effect on surrounding water protons, the signal is quantitative, background‐free, and independent of tissue depth, but it produces no anatomical image of its own [50]. Reported spatial resolution reaches 330 *μ*m in optimized preclinical systems, although most operate at 1–2 mm, and temporal resolution reaches 46 frames per second in three dimensions [50]. A single preclinical stroke study exists: In mice subjected to transient middle cerebral artery occlusion (*n* = 3), MPI measured a cerebral blood flow reduction of 89.15*%* ± 1.33*%* against 91.52*%* ± 2.76*%* by dynamic susceptibility contrast MRI [50]. Because MPI requires much weaker fields than MRI, scanners can, in principle, be built as compact resistive units, and a human‐sized low‐field brain scanner with a footprint of about one square meter has been demonstrated for stroke and intensive care monitoring, operating at 5 mm resolution and 2 Hz [50]. This should not be overstated: No human MPI imaging has been performed, no MPI‐specific tracer is approved by any regulator, and all preclinical work uses iron oxide agents repurposed off‐label [50, 53]. Bedside MPI is a plausible eventual application rather than an existing capability.

Photoacoustic imaging (PAI) detects the ultrasound waves generated when tissue absorbs a pulsed laser and undergoes transient thermoelastic expansion, combining optical absorption contrast with the depth penetration of ultrasound. It requires no ionizing radiation and uses hemoglobin as an endogenous chromophore, so vascular anatomy and oxygen saturation can be imaged without an exogenous agent, and advances in photoacoustic microscopy and CT have improved resolution and speed [57]. Exogenous NP contrast can extend PAI to molecular targets, as in the MnCO_3_@BSA‐ICG construct above [47]. The principal limitation for stroke is depth: Transcranial imaging of the adult human brain through intact skull remains unsolved, and published stroke applications are confined to rodents [57].

### 5.4. Nanosensors and Biomarkers

Where advanced imaging is unavailable or where triage must occur before imaging, blood biomarkers offer an alternative. Many candidates have been identified, but clinical adoption has been limited by the absence of assays fast and sensitive enough for the prehospital or emergency setting, since laboratory enzyme‐linked immunosorbent assays require hours and dedicated instrumentation [58]. Nanomaterial‐based biosensors provide a large surface area for analyte capture and transduce binding directly into an optical or electrochemical signal, enabling point‐of‐care formats [58].

Biomarkers can help distinguish hemorrhagic from ischemic stroke: Glial fibrillary acidic protein (GFAP) rises within a few hours of hemorrhagic stroke, whereas in ischemic stroke, serum levels peak only at 48–72 h [59]. Others indicate etiology, such as N‐terminal pro‐B‐type natriuretic peptide (NT‐proBNP) in cardioembolic stroke [60]. A fiber‐optic nanogold‐linked immunosorbent assay (FONLISA) using GNPs in a sandwich format detected NT‐proBNP over a linear range of 0.50–5000 pg/mL with a limit of detection of 0.058 pg/mL; that limit was established with standards in buffer, matrix effects were assessed in spiked serum, and clinical correlation was demonstrated against a commercial analyzer in 13 human plasma specimens [52]. Optical biosensors are also being developed for GFAP: Lanthanide‐doped upconversion NPs conjugated to an anti‐GFAP antibody gave a near‐linear photoluminescence quenching response across 5–5000 pg/mL in human serum [51]. These are analytical proof‐of‐concept studies. None has been evaluated prospectively in an unselected population with suspected stroke, and the limits of detection quoted were determined in buffer rather than whole blood [51, 52, 58]. The accuracy such a test would need is also worth stating, because it is demanding: To be acted on before imaging, it would have to exclude hemorrhage with a negative predictive value approaching that of noncontrast CT, and to be useful for prehospital triage, it would have to identify large‐vessel occlusion well enough to justify bypassing the nearest hospital. No nanomaterial biosensor has yet been tested against either standard.

## 6. Therapeutic Applications

### 6.1. Drug Delivery

Drug delivery is the most developed therapeutic application, and Table 2 summarizes the platforms, advantages, and translational barriers, with study‐level evidence in Table 3. Wu et al. developed AMD3100‐conjugated shrinkable poly(2,2 ^′^‐thiodiethylene 3,3 ^′^‐thiodipropionate) nanoparticles (ASPTT NPs), which are ROS‐reactive, shrink on thrombin‐mediated cleavage of an embedded peptide linker, and carry AMD3100 to engage CXCR4 on ischemic brain endothelium. The parent NPs consumed up to 90% of hydrogen peroxide in a cell‐free assay, and AMD3100 conjugation increased accumulation in the ischemic region 2.1‐fold relative to the untargeted particle. Glyburide‐loaded ASPTT NPs given intravenously at 5 *μ*g/kg glyburide at 0, 24, and 48 h after transient middle cerebral artery occlusion in mice reduced infarct volume by 41% compared with vehicle, reduced brain water content by 11% and blood–brain barrier leakage by 15%, and improved neurological score and survival [36]. The animals were male, without comorbidity, treatment began immediately after reperfusion, safety assessment was limited to organ histology, and no bleeding or hypoglycemia endpoint was reported. PEGylated liposomes are used to encapsulate neuroprotective drugs because the PEG corona prolongs circulation and the bilayer permits sustained release [21, 23], and a broader review concludes that liposomes, lipid NPs, polymeric NPs, micelles, and biomimetic carriers can each improve drug stability and delivery to ischemic tissue, while noting that the supporting evidence is preclinical [82].

**Table 2:** Therapeutic applications of nanoparticles in stroke management, with translational barriers.

Strategy	Representative platforms	Key advantage	Key limitation or barrier	Evidence tier
Drug delivery	PEGylated liposomes [23]; mesoporous silica, ultrasound‐triggered [61]; ROS‐responsive polymer with glyburide [36]	Improved bioavailability; reduced systemic toxicity; payload protected	Delivered dose fraction small and inconsistently reported [14]; dosing at reperfusion; no large‐animal data	In vitro; small animal
Neuroprotection	CeO_2_, regenerable SOD and catalase mimic [62, 63]; SeNPs [64]; Cur@GAR NPs [39]; RES.CP NPs [65]	Catalytic, not consumed stoichiometrically; barrier stabilization	Long‐term retention of inorganic cores; transaminase rise at high ceria dose [63]; no bleeding endpoints	In vitro; small animal
Gene and RNA therapy	Ionizable lipid nanoparticles [66, 67]; VCAM‐1‐targeted LNP with IL‐10 mRNA [20]; quantum dot‐siRNA [68]	Protects labile cargo; no genome modification with mRNA; established human manufacturing route	Endosomal escape inefficient; durability uncertain; MMP‐9 evidence in vitro only	In vitro; small animal; human precedent in other diseases
Stem cell tracking	SPION‐labeled bone marrow stromal cells [69, 70]	Real‐time tracking; trophic and anti‐inflammatory effects of the cell	Label persists after cell death; intracerebral delivery in the one human study; magnetic guidance unproven	Small animal; Phase I human
Exosome therapy	Mesenchymal stem cell–derived exosomes [71, 72]	Crosses the barrier; low immunogenicity; intranasal delivery feasible	No potency assay or characterization standard; donor batch variability; leading study is a perinatal, preventive model [72]	Small animal, part extrapolated; Phase I/II registered
Thrombolysis	Discoidal shear‐responsive PLGA [25]; cRGD liposomes [37]; magnetically guided PLGA [38]; phase change with ultrasound [73]	Concentrates thrombolytic at the clot; dose sparing; shorter bleeding times than free drug [25, 73]	Two key studies noncerebral [38, 73]; one in vitro in sheep blood [37]; interaction with thrombectomy unstudied	In vitro; small animal
Hemorrhagic stroke	PEG‐carbon clusters [74]; BSA‐SeNPs on Nrf2/GPX4 [75]; proefferocytic liposomes [76]	Targets a mechanism with clinical precedent; 44%–69% hematoma reduction with intravenous dosing [20, 76]	No registered trial in any hemorrhagic subtype; deferoxamine precedent not positive at 90 days [77]; no replication	Small animal only

*Note:* Evidence tiers are defined in Section 2 of the manuscript.

Abbreviations: BBB, blood–brain barrier; CeO2, cerium oxide; Cur@GAR NPs, curcumin‐loaded gelatin nanoparticles modified with the RVG29 peptide; IL‐10, Interleukin 10; LNP, lipid nanoparticle; MMP‐9, Matrix Metalloproteinase 9; PLGA, poly(lactic‐co‐glycolic acid); RES.CP NPs, resveratrol‐loaded copolymer (PLGA) nanoparticles; ROS, reactive oxygen species; SeNPs, selenium nanoparticles; SOD, superoxide dismutase; SPION, superparamagnetic iron oxide nanoparticle; VCAM‐1, Vascular Cell Adhesion Molecule‐1.

**Table 3:** Comparative evidence for therapeutic nanoparticle platforms in stroke. Values were read from the primary publication. NR, not reported; FO, reported only as a figure, so no value is quoted.

Platform and payload	Model and species	Size/zeta	Route, dose, window	Main outcome	Safety assessed	Readiness
ASPTT NPs; glyburide [36]	1.5 h tMCAO, male mice	NR/NR	IV, 5 *μ*g/kg, 0, 24, 48 h; at reperfusion	Infarct, −41%; brain water, −11%; BBB leakage, −15%; brain uptake 2.1x	Organ histology	Small animal
NBP‐CeO2 NPs; butylphthalide [62]	MCAO/R, mice	24–36/+6.2 to +3.9	IV, 0.4 mg/kg daily; at reperfusion	Infarct 39.8%–10.6% (bare ceria 20.6%); water 81.3%–78.4%	Organ histology	Small animal
MMTCe, membrane‐cloaked ceria [63]	90 min tMCAO, male rats	122/−9.3	IV, 1.5 mg/kg Ce, 0, 24, 48 h; at reperfusion	Infarct ~50%–17.1*%* ± 2.3*%*; water to 72.4%; CD31 area 9.2%–27.8%	Blood, histology; ALT and AST raised at high dose	Small animal
HD@iNanoAOX; hirudin [42]	Permanent photothrombotic, male mice	65.2 ± 3.0/−7.8	IV, 1.0 mg/kg, single; 10 min after occlusion	Hirudin half‐life 21.9–91.5 min; infarct and edema reduced (FO); free hirudin increased subarachnoid bleed	Cerebral hemorrhage area only	Small animal
Platesome‐DFO; deferoxamine [22]	60 min tMCAO, male mice	136.6 ± 1.6/~−20	IV, 25 mg/kg, single; at reperfusion	Brain iron normalized; GPX4 up, ACSL4 and MDA down; infarct smaller (FO)	Histology, blood, hemolysis; PT, aPTT, TT normal	Small animal
RvD2 nanovesicles [78]	60 min tMCAO, male mice	190/−10 to −14	IV, 8 *μ*g/kg, single; 1 h after reperfusion	Infarct 16% vs. 46%; neutrophil infiltration −10‐fold	In vitro viability only	Small animal; xenogeneic membrane
CS15‐GNPs, ApoE‐mimetic gold [27]	90 min tMCAO, male rats	38.1 ± 1.7/−4.5	Intranasal, 3.5 or 7 *μ*g/kg; before reperfusion	Infarct 21.2*%* ± 3.9*%* to 10.8*%* ± 4.6*%*	7‐day repeat‐dose: Blood, organ and nasal histology	Small animal
circSCMH1@LNP1 [67]	Permanent photothrombotic, male mice	140–170/−1 to −3	Intranasal, 12–120 *μ*g/kg; 24 h after stroke	Sensorimotor and maze gains to Day 28; no infarct measured	T cells, bloods, organ and nasal histology, brain cytokines	Small animal; realistic window
RES.CP NPs; resveratrol [65]	tMCAO, male rats	~30/−38	IV, 20 mg/kg, single; at reperfusion	NF‐*κ*B p65 down (*p* = 0.0006); TNF‐*α* down, IL‐10 up (FO); no infarct measured	None	Small animal
Discoidal PLGA; tPA [25]	Embolic MCAO, mice	FO/FO	IV, 1 mg/kg tPA; NR	Bleeding time 16.9 ± 1.6 min with free tPA vs. 4.7 ± 0.7 min with conjugate	Bleeding time, PT, aPTT, fibrinogen, histology	Small animal
pPMNP‐rtPA, magnetic PLGA [38]	Hind‐limb iliac embolism, male rats	321 ± 27/−22	Intra‐arterial, 0.3 U/kg with 0.5 T magnet; 5 min	Reperfusion at 35 vs. 60 min at 20% of the free dose	Cytotoxicity, hemolysis; bleeding not measured	Small animal; not a cerebral model
PPrC NPs with ultrasound; rtPA [73]	FeCl_3_ jugular vein thrombosis, rats	209 ± 3/−6.4	IV then focused ultrasound 1 W/cm^2^, 30 min	Residual thrombus 17.7% vs. 46.3%	Histology, bloods; bleeding time and coagulation normal	Small animal; venous model
cRGD liposomes; tPA [37]	In vitro; sheep blood	164.6 ± 5.3/−33 to −40	Not applicable	Over 90% tPA release in 1 h on activated platelets; t50 48 vs. 122 min	None	In vitro
tPA‐PEG‐PLGA NPs [79]	In vitro; human clots	276 ± 28/−11.9	Not applicable	Clot digestion 44.8*%* ± 2.7*%*; activity 2–6x free tPA	A549 cytotoxicity only	In vitro; loading precludes therapeutic dosing
Edaravone mPEG‐b‐PLGA micelles [80]	In vitro; SH‐SY5Y under OGD	155 ± 2.5/−15	Not applicable; pretreatment	Viability 78%–84% vs. ~50% death; no infarct measured	In vitro only	In vitro
Proefferocytosis neutrophil liposomes [76]	Autologous blood ICH, male mice	~50/NR	IV, daily	Hematoma 7.04–3.96 mm^3^ (−44%); erythrophagocytosis 28.5%–67.6%	Reported in source	Small animal
VCAM‐1‐targeted LNP; IL‐10 mRNA [20]	Collagenase ICH, male mice	NR	IV, 5 *μ*g mRNA x4; 30 min	Hematoma −69%; grid walk +65%; brain delivery 4x	Reported in source	Small animal
Ceria nanoparticles [81]	Perforation SAH, rats	~3/NR	IV, 0.5 mg Ce/kg, single; 1 h	14‐day survival 88.2% vs. 21.1%; Garcia score 12.1 vs. 4.4	Reported in source	Small animal; not replicated

Abbreviations: ICH, intracerebral hemorrhage; MCAO/R, middle cerebral artery occlusion with reperfusion; OGD, oxygen–glucose deprivation; SAH, subarachnoid hemorrhage; tMCAO, transient middle cerebral artery occlusion.

Nanocarriers also permit codelivery of two agents from a single particle, so a thrombolytic and a neuroprotective or anti‐inflammatory drug can be released together or in sequence, addressing occlusion and the downstream injury cascade at once [61, 82]. Direct head‐to‐head demonstrations that a coloaded particle outperforms the same two drugs given separately remain uncommon, so this should be regarded as a design rationale supported by mechanism rather than an established result. Externally triggered release adds a further level of control: Mesoporous silica NPs release payload on exposure to focused ultrasound, liberating drug only within the insonated volume [61].

### 6.2. Neuroprotection

Mitochondrial injury and oxidative stress are principal mechanisms of reperfusion injury: Restoration of oxygen to ischemic tissue drives a burst of ROS from damaged electron transport chains, overwhelming endogenous antioxidant defenses and propagating lipid peroxidation, blood–brain barrier breakdown, and cell death [62, 83]. Cerium oxide cycles between Ce^3+^ and Ce^4+^ and acts as a regenerable mimic of superoxide dismutase and catalase, so a single particle can scavenge ROS repeatedly rather than stoichiometrically [83]. Li et al. loaded dl‐3‐*n*‐butylphthalide onto cerium oxide nanoparticles (NBP‐CeO_2_ NPs) (hydrodynamic diameter increasing from approximately 24 to 36 nm on loading). Given intravenously at 0.4 mg/kg daily from reperfusion, they reduced infarct volume from approximately 39.8% to 10.6% at 3 days and brain water content in the infarcted hemisphere from approximately 81.3% to 78.4%, scavenged around 72.3% of ROS in hippocampal neurons in vitro, and improved sensorimotor and spatial learning performance over 27 days [62]. Two qualifications apply: These are approximate values read from figures with *n* = 4 for the infarct measurement, and the 10.6% infarct belongs to the drug‐loaded construct, whereas unloaded cerium oxide gave approximately 20.6%, so the effect is not attributable to ceria alone. Related constructs include ceria nanoenzymes directed at mitochondria during reperfusion injury [84] and a microglial membrane‐cloaked, triphenylphosphine‐functionalized ceria system carrying SPIO, which in rats treated at reperfusion reduced infarct volume to 17.1*%* ± 2.26*%* and brain water content to 72.37*%* ± 0.88*%* and increased CD31‐positive vessel area from 9.17*%* ± 1.72*%* to 27.75*%* ± 2.64*%*; in that study, serum transaminases were slightly raised at the highest dose, which the authors interpret as possible hepatotoxicity, and the in vivo magnetic field parameters are not reported [63]. Selenium NPs are also potent antioxidants that reach the brain and reduce neuronal death by increasing antioxidant enzyme expression and inhibiting apoptosis [64].

Natural compounds have been reformulated as NPs to overcome poor aqueous solubility and low permeability, curcumin being the standard example [39]. Yang et al. prepared curcumin‐loaded gelatin NPs and surface‐modified them with RVG29, coupling it through aldoxylated hyaluronic acid to give Cur@GAR NPs; the unmodified particle is Cur@Gel NPs. The modified particles showed greater neuronal uptake and antioxidant activity (DPPH scavenging 65.67*%* ± 1.53*%* against 58.67*%* ± 0.58*%*), reduced neuroinflammation and apoptosis, and improved behavioral recovery, with Morris water maze escape latency of 53 ± 8.89 s against more than 120 s in untreated animals and 28 ± 7.55 s in normal animals [39]. These are short‐term preclinical results without clinical validation. Resveratrol formulated in copolymer nanoparticles (RES.CP.NPs) (PLGA particles of approximately 30 nm, zeta potential near −38 mV) given intravenously at 20 mg/kg after occlusion in rats lowered brain nuclear factor kappa‐light‐chain‐enhancer of activated B cells (NF‐*κ*B) p65 and tumor necrosis factor alpha (TNF‐*α*), raised Interleukin‐10 (IL‐10), and improved neurological score at 24 h [65]; that study measured protein levels rather than pathway activity, reported no infarct volume or blood–brain barrier data, and performed no toxicity assessment. Such particles may additionally help preserve barrier integrity by limiting neuroinflammation [83].

### 6.3. Gene and RNA‐Based Therapy

Nucleic acid therapeutics cannot reach the brain unaided, for four reasons that a nanocarrier addresses simultaneously. Unmodified RNA is degraded within minutes by serum and tissue nucleases; as a large polyanion, it cannot cross an anionic lipid bilayer; it is rapidly cleared renally and by the mononuclear phagocyte system; and unshielded nucleic acid is recognized by innate immune sensors including Toll‐Like Receptors 3, 7, and 8 and can provoke an interferon response [66]. Encapsulation shields the cargo from nucleases and immune sensing, masks its charge, prolongs circulation, and delivers it into the endosome, from which ionizable lipids permit escape into the cytosol; surface ligands can then direct the particle to cerebral endothelium [20, 66]. Small interfering RNA (siRNA) silences genes pathologically upregulated after stroke, and antisense oligonucleotides act analogously by binding target messenger RNA; both require a carrier [11, 66].

Messenger RNA therapy instructs cells to produce a therapeutic protein rather than suppressing one and does not integrate into the host genome, avoiding the insertional mutagenesis risk of DNA‐based gene therapy [66]. Preclinical work supports the approach. In vitro, quantum dot‐siRNA nanoplexes silencing Matrix Metalloproteinase‐9 (MMP‐9) preserved tight junction protein expression and barrier integrity in a cell culture model of the blood–brain barrier, although no animal work was performed and no measure of brain swelling was made [68]. In vivo, Vascular Cell Adhesion Molecule‐1‐targeted lipid NPs carrying IL‐10 messenger RNA reached the brain approximately four times more efficiently than untargeted particles, reduced hematoma volume by approximately 69%, and improved motor performance by approximately 65% in a mouse model of intracerebral hemorrhage [20]. Circular RNA offers greater intrinsic stability than linear messenger RNA because it lacks free ends for exonuclease attack: circSCMH1 encapsulated in an SM‐102 lipid NP (140–170 nm, encapsulation efficiency above 90%) and given intranasally at 120 *μ*g/kg 24 h after photothrombotic stroke in mice improved sensorimotor and spatial learning performance to 28 days, increased vessel density and myelin basic protein, and reduced glial activation, with no change in peripheral T‐cell subsets, serum biochemistry, or nasal mucosal histology [67]. This is one of the few studies using a clinically plausible delayed window, although it reported no infarct volume. Delivery of messenger RNA encoding vascular endothelial growth factor to promote angiogenesis has been proposed but not demonstrated in a stroke model, and CRISPR/Cas9 components are large and require a nanocarrier for brain delivery, with no stroke application yet at in vivo proof of concept [83].

### 6.4. Stem Cell and Exosome‐Mediated Therapies

Nanoengineered stem cells are stem cells whose surface or cytoplasm has been modified with NPs, either to make them trackable by imaging, to steer them with an external field, or to load them with a cargo they would not otherwise carry; the cell provides the biology, and the NP provides tracking or control [69]. Mesenchymal stem cells are the usual substrate because they are immunomodulatory and secrete trophic factors, but their benefit depends on reaching and remaining at the injured tissue, which is inefficient after systemic delivery [69]. Labeling with SPIO NPs allows cells to be tracked by MRI [69]. This is the one cell‐based nanotechnology in stroke with human data: In the RAINBOW trial, autologous bone marrow stromal cells labeled with ferucarbotran were transplanted intracerebrally in patients with subacute ischemic stroke and tracked by MRI [70].

Exosomes are endogenous extracellular vesicles of approximately 30–150 nm that carry protein and RNA cargo between cells, can cross the blood–brain barrier, and are being investigated as naturally derived nanocarriers with low immunogenicity; they can be loaded with therapeutic cargo or hybridized with synthetic NPs to add imaging or targeting functions [71]. The best‐known intranasal exosome study in brain injury was conducted in Postnatal Day 2 rat pups subjected to systemic lipopolysaccharide, permanent carotid cauterization, and hypoxia, with exosomes given before the ischemic insult; survival rose from 23.2% to 41.2%, and myelin and neuronal markers were partially preserved, although long‐term memory was not rescued [72]. That is a model of perinatal brain injury with preventive dosing and is cited as extrapolated evidence of intranasal exosome delivery rather than evidence in adult stroke. Exosome therapy nevertheless has the strongest clinical footing among nanoscale platforms in stroke, since allogeneic mesenchymal stem cell–derived exosomes enriched with miR‐124 have been registered for a Phase I/II study in acute ischemic stroke (Table 4).

**Table 4:** Registered clinical studies of nanoparticle‐related interventions in stroke, from ClinicalTrials.gov, the EU Clinical Trials Register, UMIN‐CTR, and the Iranian Registry of Clinical Trials. No registered study of a nanoparticle therapeutic in intracerebral or subarachnoid hemorrhage was identified.

Registration	Agent or platform	Indication	Design and n	Phase and status	Sponsor
NCT02963376 [85]	Dodecafluoropentane emulsion, perfluorocarbon nanodroplets	Acute ischemic stroke	Randomized dose‐escalation, 24	Ib/II, completed 2018	NuvOx, United States
NCT03384433	Mesenchymal stem cell exosomes with miR‐124	Acute ischemic stroke	Randomized, placebo‐controlled, 5	I/II, registered 2019	Isfahan Univ Med Sci, Iran
UMIN000026130 [70]	Bone marrow stromal cells labeled with SPIO	Subacute ischemic stroke	Open‐label dose–response, 6–10	I, completed	Hokkaido Univ, Japan
NCT03084523	Ferumoxytol	Intracranial atherosclerosis and stroke risk	Imaging comparison vs. gadolinium, 30	I, completed 2024	Yale, United States
NCT02452216	Ferumoxytol‐enhanced MRI	CNS conditions including stroke	Single‐arm imaging, 10	Early I, completed 2017	Stanford, United States
EudraCT 2020‐002059‐38 [86]	ApTOLL aptamer; nanoscale but non‐particulate	Acute ischemic stroke with thrombectomy	Randomized, placebo‐controlled, 151	1/2, completed 2022	AptaTargets, EU
IRCT20120215009014N214 [87]	Oral nanocurcumin 80 mg daily	Ischemic stroke	Randomized placebo‐controlled pilot, 40	Pilot, published 2025	Guilan Univ Med Sci, Iran

Abbreviations: CNS, central nervous system; SPIO, superparamagnetic iron oxide.

## 7. Nanotechnology in Rehabilitation and Monitoring

Wearable devices built from nanostructured materials are being developed for poststroke rehabilitation and monitoring. A triboelectric nanogenerator (TENG) converts mechanical motion into an electrical signal through contact electrification combined with electrostatic induction: Two dissimilar materials are brought into contact and separated, transferring charge between their surfaces, and the resulting potential difference drives electrons through an external circuit, so limb movement generates a voltage without a battery [88]. The dependence on nanotechnology is not incidental: Output scales with effective contact area and surface charge density, so surfaces are patterned at the nanoscale with nanowires, nanopillars, or NP coatings to raise both, which is what makes a self‐powered device sensitive enough to resolve the small, slow movements of a hemiparetic limb [88]. Such devices have been configured as self‐powered wearable sensors that monitor upper‐limb movement and support home‐based rehabilitation [89]. Nanomembrane sensor systems use a related principle: Ultrathin electrodes conform to skin without adhesive, and because their thickness is on the order of hundreds of nanometers, they follow skin deformation rather than resisting it, preserving signal quality during movement and allowing combined sensing and haptic feedback in a glove format. These were developed in patients with spinal cord injury and would need separate evaluation in stroke, where the pattern of sensorimotor loss differs [90].

Injectable hydrogels approximating the mechanical and biochemical properties of brain extracellular matrix are being developed to fill the infarct cavity and support regeneration. Jian et al. embedded polyelectrolyte complex NPs carrying Stromal Cell–Derived Factor 1 alpha and basic fibroblast growth factor within a hyaluronic acid hydrogel, the chemokine carrier linked through a matrix metalloproteinase‐cleavable peptide and the growth factor carrier through a noncleavable one, and injected the construct into the cavity 7 days after photothrombotic stroke in rats. The combination increased doublecortin‐positive neuroblasts and vascular density relative to hydrogel alone and outperformed a bolus of the same growth factors, which never separated from vehicle on motor testing. Its advantage over hydrogel alone was confined to those histological measures: At Day 28, impaired forelimb use was 46.9*%* ± 3.8*%* with the loaded construct against 45.7*%* ± 0.5*%* with plain hydrogel and 34.4*%* ± 5.2*%* with vehicle, and both hydrogel groups reached baseline by Day 35, so the functional benefit is attributable to the scaffold rather than the NPs [91]. The approach also requires craniotomy and surgical removal of necrotic tissue, placing it in a different clinical category from systemic agents.

## 8. NPs in Hemorrhagic Stroke

### 8.1. Why Hemorrhagic Stroke Is a Different Delivery Problem

Most of the NP literature concerns ischemic stroke, and platforms optimized for it do not transfer unaltered. After intracerebral hemorrhage, injury proceeds in phases: mass effect and clot retraction within hours, then thrombin‐ and complement‐mediated injury as the coagulation cascade activates, and then erythrocyte lysis over days with release of hemoglobin and free iron, with perihematomal edema developing over the first 24 h and peaking between Days 3 and 7 [92]. Free iron drives lipid peroxidation and ferroptosis, an iron‐dependent form of regulated cell death; in rodent intracerebral hemorrhage, the Ferroptosis Inhibitor Ferrostatin‐1 reduced lesion volume, iron deposition, and neurological deficit [93]. Clearing the hematoma is itself therapeutic: Microglial and macrophage phagocytosis of erythrocytes is driven by Nuclear Factor Erythroid 2–Related Factor 2 (Nrf2) acting through CD36, and Nrf2‐null animals clear hematoma more slowly [94]. Two features matter for design. The therapeutic target is often the phagocyte rather than the neuron, which changes the desired biodistribution. And the blood–brain barrier around a hematoma is disrupted, whereas the hematoma itself is a poorly perfused mass, so a particle circulating in blood does not necessarily reach the tissue that needs it.

### 8.2. NP Approaches Tested in Intracerebral and Subarachnoid Hemorrhage

Iron chelation and catalytic antioxidant strategies dominate. PEGylated hydrophilic carbon clusters, catalytic superoxide dismutase mimics, reduced haem‐induced oxidative genomic and mitochondrial DNA damage in rodent intracerebral hemorrhage, and a deferoxamine‐conjugated derivative combining chelation with ROS scavenging outperformed the unconjugated particle [74]. Bovine serum albumin–stabilized selenium NPs of approximately 40 nm reduced the hemorrhagic area from 4.39% to 1.94% at the higher dose, preserved hippocampal neurons, improved novel object recognition, and upregulated the Nrf2 and Glutathione Peroxidase 4 axis [75]. The two most quantitatively convincing results target hematoma clearance directly and use intravenous dosing. Neutrophil membrane–coated, acid‐responsive liposomes codelivering an ADAM17 inhibitor and a liver X receptor agonist reduced Day‐3 hematoma volume from 7.04 ± 0.19 to 3.96 ± 0.52 mm^3^, approximately 44%, while raising the proportion of erythrocyte‐containing microglia and macrophages from 28.5% to 67.6% [76]. VCAM‐1‐targeted lipid NPs carrying IL‐10 messenger RNA, given intravenously at 0.5, 24, 48, and 68 h after collagenase‐induced hemorrhage, reached the brain approximately four times more efficiently than untargeted particles and produced approximately a 69% reduction in hematoma volume and approximately a 65% improvement in grid‐walk performance at 72 h [20]. In aneurysmal subarachnoid hemorrhage, where oral nimodipine remains the only guideline‐recommended agent [5], ceria NPs of approximately 3 nm given as a single intravenous dose of 0.5 mg Ce/kg 1 h after endovascular perforation in rats reduced neuronal death, macrophage infiltration, and edema at 72 h and improved 14‐day survival from 21.1% to 88.2% [81]; that effect size is exceptional and has not been independently replicated, which should temper its interpretation.

### 8.3. Hemostasis and the State of the Evidence

Hematoma growth is a modifiable determinant of outcome, but the clinical record is cautionary. Tranexamic acid reduced hematoma expansion and early death in a Phase III trial of 2325 patients without improving the primary functional outcome at 90 days [95]. Ultraearly Recombinant Factor VIIa reduced 24‐h hematoma growth by 3.7 mL but did not improve 180‐day modified Rankin Scale outcome, and life‐threatening thromboembolic complications within 4 days occurred in 15 treated participants against four controls, a relative risk of 3.41 (95% CI 1.14–10.15) [96]. Any prohemostatic NP, therefore, must reduce expansion and improve function without inducing thrombosis. Platelet‐mimetic hemostatic NPs that bind activated Glycoprotein IIb/IIIa improve survival after trauma [97], but to our knowledge, none has been tested in an intracerebral hemorrhage model, which, given the Factor VIIa thromboembolic signal, is a substantive gap rather than an oversight. More broadly, the NP literature in hemorrhagic stroke is smaller and less mature than in ischemic stroke. We identified no registered clinical trial of a NP therapeutic in either hemorrhagic subtype, or the clinical precedent for the leading mechanism is a free small molecule that was not positive on its primary endpoint: In the i‐DEF Phase II trial, deferoxamine mesylate met futility criteria for the proportion achieving a modified Rankin Scale score of 0–2 at Day 90, although a signal favoring treatment was present at Day 180 [77]. The closest clinical particle therapeutic is not a NP: EG‐1962, a sustained‐release nimodipine PLGA microparticle given intraventricularly, reached Phase III and was stopped at interim analysis for futility [98]. Several of the strongest preclinical results came from intrathecal administration; almost all use a single model, species, and sex, and there is essentially no independent replication.

## 9. Preclinical and Clinical Translation of NPs in Stroke

The distance between preclinical results and a clinical trial is best judged by asking which platforms already have clinical precedent somewhere in medicine, which remain confined to cell culture or small animals, and what the hyperacute setting specifically demands. The platforms with the strongest external precedent are lipid NPs, iron oxide, and PLGA. Lipid NPs are established human medicines for nucleic acid delivery, so formulation, manufacturing, and regulatory pathways are known [66]. Iron oxide NPs are already administered to patients, although ferumoxytol is approved only for iron deficiency anemia and all imaging use is off‐label under a boxed warning [53]. PLGA is an approved parenteral excipient with a long record in depot formulations [24], and a PLGA microparticle depot has been taken to Phase III in subarachnoid hemorrhage, where it failed on efficacy rather than safety or manufacturability [98]. By contrast, MENPs, MOFs, upconversion NPs, and most membrane‐cloaked constructs have no clinical precedent in any disease, and several exist only as single reports from single laboratories. Several thrombolytic constructs remain entirely in vitro: a tPA‐loaded PEGylated PLGA NP whose authors state that its low drug loading precludes delivery of a therapeutic dose in vivo [79] and an edaravone‐loaded micelle evaluated only in SH‐SY5Y cells under oxygen–glucose deprivation, with no animal work and no measurement of infarct volume [80]. Figure 2 summarizes the pathway from experimental development to clinical application, including the principal points of attrition.

**Figure 2:**
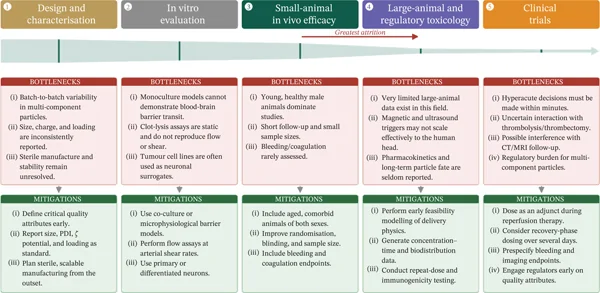
Translational pathway for nanoparticle‐based stroke therapies and the stage‐specific bottlenecks that cause attrition.

The hyperacute setting imposes constraints most preclinical designs do not model. Decisions are made within minutes of arrival, from a noncontrast scan, often before subtype, occlusion site, or medication history are known. An agent needing reconstitution, warming, slow infusion, or an external magnetic field is unlikely to fit that workflow, and magnetic guidance faces the further problem that focusing a field on a deep intracranial thrombus is not equivalent to focusing it on a rodent hind limb [38, 63]. Almost every therapeutic study in Table 3 gave the first dose at or within minutes of reperfusion, earlier than is achievable in practice; the exception, a lipid NP given intranasally at 24 h, targeted recovery rather than acute salvage [67]. Realistic trial designs would, therefore, test NPs either as adjuncts given at the time of thrombolysis or thrombectomy, when the patient is already monitored and vessel status is known, or as recovery‐phase treatments given over days, where the window problem disappears.

Combination with reperfusion therapy raises questions the current literature does not answer. The first is whether a NP given alongside alteplase or tenecteplase increases bleeding. Only a minority of the therapeutic studies reviewed here measured any bleeding or coagulation endpoint, and where they did the results were informative: Free tPA prolonged tail bleeding time from 2.8 ± 0.3 to 16.9 ± 1.6 min and disturbed prothrombin time, activated partial thromboplastin time, and fibrinogen, whereas a NP conjugate prolonged bleeding time only to 4.7 ± 0.7 min and left coagulation indices near sham values [25], a perfluorocarbon thrombolytic nanoplatform likewise showed bleeding times markedly shorter than free recombinant tPA with normal coagulation indices [73], and a platelet membrane–cloaked liposome carrying deferoxamine left prothrombin, activated partial thromboplastin, and thrombin times unchanged [22]. Elsewhere, claims of reduced bleeding risk are inferences from dose sparing rather than measurements [38]. The second question is whether the agent interferes with follow‐up imaging. This is not hypothetical: Iron oxide NPs produce marked T2 and T2∗ signal loss persisting for days, which can be mistaken for or obscure hemorrhagic transformation on gradient‐echo and susceptibility‐weighted sequences, and high atomic number contrast agents attenuate X‐rays sufficiently to mimic blood on noncontrast CT, precisely the discrimination on which postthrombolysis management depends [48, 49, 53]. Any agent proposed for use around reperfusion therapy should report its effect on follow‐up imaging, and none of the studies reviewed here does so. A third question is interaction with the thrombus itself: A particle binding activated platelets or fibrin may alter clot mechanics and, therefore, the success of stent retriever or aspiration thrombectomy, which has not been studied.

A quieter obstacle is that the pharmacokinetic data needed to design a human study mostly do not exist. Of the therapeutic constructs reviewed here, few report a plasma concentration–time curve, a circulating half‐life, an area under the curve, or a quantitative measure of the fraction of the administered dose reaching brain tissue; brain delivery is most often demonstrated by fluorescence imaging of a lipophilic dye, which reports where the label went rather than how much drug arrived and cannot distinguish intravascular from parenchymal signal. Where pharmacokinetics were measured, they were sometimes obtained in healthy rather than stroke animals, so the influence of a disrupted barrier on disposition is unknown [42]. Long‐term fate is reported still less often, which matters most for nonbiodegradable inorganic cores, where organ retention rather than acute toxicity is the plausible risk [13, 99]. Without these data, a first‐in‐human dose cannot be justified by allometric scaling, and a regulator has no basis on which to set a starting dose or a monitoring schedule [16].

Patient selection and manufacturing complete the picture. Effects that depend on leaky vasculature will vary with the timing and degree of barrier disruption, which differs between large‐vessel and lacunar stroke and between patients [34]. Older, hypertensive and diabetic patients, who constitute most of the treated population, are almost absent from the preclinical record. The constructs closest to clinical use are also the simplest: Multicomponent particles combining a biological membrane, an inorganic core, a targeting ligand, and a drug payload present compounded challenges of batch‐to‐batch reproducibility, sterilization, stability, and characterization, and the regulatory expectation is that every attribute affecting quality and performance is controlled and justified [16, 32].

Several barriers are common to the field. Biocompatibility and long‐term toxicity remain the primary concern, particularly for nonbiodegradable inorganic materials, which has prompted interest in biodegradable alternatives [99]. Toxicity is not a property of a material in the abstract but of a particular particle at a particular dose by a particular route, depending on size, shape, surface chemistry, aggregation state, dissolution, and clearance [15, 26]. The safety packages in the studies reviewed here are thin: Most report organ histology after a single dose, several report serum biochemistry, few report hematology or body weight, almost none report immunogenicity or repeat‐dose toxicity, and one ceria construct showed a rise in transaminases at its highest dose that its authors interpreted as possible hepatotoxicity [63]. Constructs built from xenogeneic or allogeneic cell membranes, including human leukemia cell membranes given to mice and human platelet membranes used in murine imaging, carry immunogenicity questions that have not been addressed [32, 49, 78]. The complexity of human stroke, in which comorbidities are the rule, is poorly reproduced in young healthy animals, and this mismatch is a recognized contributor to translational failure [9, 10]. Particles smaller than approximately 6 nm are cleared renally, whereas larger particles must be biodegradable to avoid accumulation [13, 100]. Regulatory approval requires pharmacokinetic and safety data that are demanding to generate for multicomponent systems in a disease as time‐critical and heterogeneous as stroke [16].

### 9.1. Registered Clinical Studies of NP‐Related Interventions in Stroke

To establish how far the field has reached patients, ClinicalTrials.gov, the EU Clinical Trials Register, UMIN‐CTR, and the Iranian Registry of Clinical Trials were searched for registered studies of NP or nanoscale interventions in ischemic or hemorrhagic stroke. We identified seven registrations, summarized in Table 4. Only one therapeutic NP, a dodecafluoropentane perfluorocarbon nanodroplet emulsion acting as an oxygen carrier, has completed a randomized controlled dose‐escalation study in acute ischemic stroke. That trial enrolled 24 participants and was designed to establish a maximum tolerated dose rather than efficacy (NCT02963376) [85]. The remainder are an allogeneic mesenchymal stem cell–derived exosome preparation enriched with miR‐124 in a five‐participant Phase I/II study (NCT03384433); the RAINBOW Phase I trial of autologous bone marrow stromal cells labeled with SPIO for magnetic resonance tracking (UMIN000026130) [70]; two imaging studies using ferumoxytol, one comparing vessel wall enhancement against gadolinium in intracranial atherosclerosis and one imaging central nervous system inflammation in a cohort including stroke (NCT03084523, NCT02452216); a Phase I/II trial of ApTOLL, a Toll‐Like Receptor 4 antagonist aptamer and, therefore, a nanoscale oligonucleotide rather than a particle, included for completeness (EudraCT 2020‐002059‐38) [86]; and a 40‐participant pilot randomized trial of oral nanocurcumin 80 mg daily for 1 month, which reported lower National Institutes of Health Stroke Scale and modified Rankin Scale scores and lower serum Interleukin‐6, with no change in infarct volume (IRCT20120215009014N214) [87]. Two absences are as informative as the entries: The EU Clinical Trials Register returned no trial combining stroke with either NP or liposome, and no registered trial of a NP therapeutic in intracerebral or subarachnoid hemorrhage was identified. NP‐based therapy in stroke, therefore, remains investigational, and the clinical experience that exists is concentrated in diagnostic and cell‐tracking applications of iron oxide, reflecting the fact that those materials were already approved for another indication.

It follows that the first clinical studies in this field should be designed around the constraints identified above rather than around the platform. Three designs appear feasible now. An adjunctive design would enroll patients with large‐vessel occlusion already proceeding to thrombectomy; administer the agent at the time of arterial access when the diagnosis, the occlusion site, and the concomitant thrombolytic are all known; and take reperfusion grade and 24‐h hemorrhagic transformation as coprimary safety endpoints, with final infarct volume on Day 5 imaging as the efficacy signal; this suits antioxidant and anti‐inflammatory constructs aimed at reperfusion injury. A recovery‐phase design would enroll patients days after stroke and give repeated doses over weeks, with a 90‐day modified Rankin Scale distribution as the primary endpoint; this suits nucleic acid and regenerative platforms and removes the hyperacute window problem entirely, as the delayed intranasal circular RNA work illustrates [67]. A diagnostic design would compare a NP contrast agent against the current standard in the same patients, with a prespecified reference standard, and is the most immediately achievable because the comparator is well defined and the agent need not show a clinical benefit to be useful. All three require what is currently missing: prespecified bleeding endpoints, prespecified assessment of interference with follow‐up imaging, and a defensible starting dose.

## 10. Future Directions

The most useful next steps are methodological rather than chemical. Four categories of study would change what we can conclude. First, replication and dose‐ranging: Many results in Table 3 are single reports from single laboratories, several with group sizes below six, and multilaboratory replication would establish which effects are real before further formulation work. Second, models that resemble patients: aged animals, both sexes, and animals with hypertension, diabetes, or hyperlipidemia, using randomization, allocation concealment, blinded outcome assessment, and prespecified sample sizes in accordance with established preclinical recommendations [10]. Third, clinically realistic dosing: delayed windows of at least 3–6 h, a route achievable in an emergency department, and follow‐up to at least 28 days with functional rather than purely histological endpoints. Fourth, safety studies designed around the questions clinicians will ask: bleeding time and coagulation indices in every study of an agent intended for use alongside thrombolysis, the effect of the agent on follow‐up CT and MRI, repeat‐dose and immunogenicity testing for membrane‐cloaked constructs, and quantification of long‐term tissue retention for nonbiodegradable inorganic cores. Head‐to‐head comparison between lipid, polymeric, and inorganic platforms in a common model would allow the field to prioritize, which it currently cannot do. Beyond this, artificial intelligence applied to imaging and clinical data may help identify the stroke subtypes and time windows in which a given platform is most likely to work, supporting enrichment in early‐phase trials. Combining NPs with regenerative scaffolds may support neurorestoration in the recovery phase, where the window is measured in weeks, and translational barriers are correspondingly lower; nanobiosensors may find their first clinical role in prehospital triage rather than treatment.

## 11. Study Limitations, Strengths, and Implications

Two sets of limitations should be distinguished. Regarding the evidence base, underreporting of negative findings contributes to publication bias and inflates apparent efficacy. The studies summarized in Table 3 are almost uniformly conducted in young, male, comorbidity‐free rodents, treated within minutes of the insult, followed for 24 h to 28 days, with group sizes between three and 15 and with randomization and blinding infrequently reported; a substantial proportion report their principal outcomes only graphically, so effect sizes cannot be extracted. Safety remains a concern, particularly for inorganic and metallic NPs that may accumulate in the liver, spleen, and brain and whose long‐term degradation and clearance are incompletely characterized. At the same time, organic systems requiring extensive surface modification can show altered pharmacokinetics and unpredictable biodistribution. Cost, manufacturing complexity, and scalability further impede translation, and multifunctional or stimuli‐responsive particles often depend on intricate, poorly standardized synthesis.

This review has its own limitations. It is narrative rather than systematic: The search strategy is reported in full, and inclusion criteria were prespecified, but screening was not performed in duplicate with stage‐by‐stage record‐keeping, no formal risk‐of‐bias instrument was applied, and no quantitative synthesis was attempted, so selection bias in the choice of illustrative studies cannot be excluded. The search covered three databases and English‐language publications only and excluded conference abstracts, which may compound publication bias in a field where negative results are seldom published in full. Registry searching was confined to four registries and to records identifiable by NP‐related terms, so a nanoformulated agent registered under a proprietary name alone could have been missed. The tables report parameters as stated in each primary publication and record unreported values as such rather than imputing them, so they are uneven by construction. The field moves quickly, and the search closed in July 2025.

The strengths follow from the same design choices. We verified every quantitative value, chemical formula, and particle dimension against the primary publication rather than an intermediate secondary source, and we corrected several discrepancies identified in that process. Each cited study carries an explicit evidence tier, and evidence extrapolated from oncology, tissue engineering, or perinatal brain injury is labeled as such, so readers are not left to infer how much of the technology has been validated in stroke. Ischemic and hemorrhagic stroke are treated separately. The tables record model, species, route, dose, treatment window, particle size and surface charge, safety assessment, and follow‐up alongside efficacy and report translational barriers alongside advantages, allowing platforms to be compared on readiness rather than promise. Registered clinical studies are enumerated rather than asserted. The implications are practical. For clinicians, no NP therapy is close to changing stroke care, and the clinical experience with nanoscale agents is currently in imaging and cell tracking rather than treatment. For translational researchers, the constraint is no longer the availability of promising constructs but the quality and clinical relevance of the evidence supporting them. For journals and funders, requiring reporting of bleeding endpoints, imaging interference, and evidence tier would materially improve the interpretability of this literature.

## 12. Conclusion

Lipid‐based, polymeric, inorganic, and biomimetic NPs have been developed to address the delivery, targeting, and toxicity limitations of conventional stroke therapy, and preclinical evidence indicates that they can improve imaging contrast, concentrate drug within ischemic and perihematomal tissue, scavenge ROS, deliver nucleic acids to the brain, and support cell tracking. That evidence is almost entirely preclinical. Efficacy was demonstrated in young, male, comorbidity‐free rodents treated within minutes of the insult and followed for a month or less, bleeding and coagulation endpoints were measured in a minority of studies, and the effect of these agents on follow‐up imaging, on thrombus mechanics, and in patients with the comorbidities that characterize real stroke populations is essentially unstudied. We identified seven registered clinical studies, only one randomized trial of a therapeutic NP, and none in hemorrhagic stroke. The platforms with the clearest path forward are those whose materials already have clinical precedent, principally lipid NPs, iron oxide, and PLGA, and the applications closest to patients are diagnostic and cell‐tracking rather than therapeutic. Realizing the therapeutic potential of these platforms will depend less on further formulation innovation than on evidence generated in models and under conditions that resemble clinical practice, on transparent reporting of safety and negative findings, and on manufacturing that can be reproduced at scale.

## Author Contributions

Conceptualization: M.F., S.B.R., M.U.H., and P.K. Data Curation: M.Q.Q., F.H., H.A., and S.Y. Investigation: M.F., M.Q.Q., F.H., S.Sa., and S.So. Project administration: M.F. and M.U.H. Resources: T.P. Supervision: M.F., S.B.R., M.U.H., M.K., B.B., T.P., and P.K. Validation: S.B.R., M.U.H., M.K., B.B., and P.K. Visualization: S.Y. and S.So. Writing—original draft: M.F., M.Q.Q., F.H., H.A., and S.Y. Writing—review and editing: M.F., H.A., S.So., S.Sa., S.B.R., M.K., B.B., T.P., and P.K.

## Funding

No funding was received for this research.

## Ethics Statement

Ethics approval was not required for this article.

## Consent

The authors have nothing to report.

## Conflicts of Interest

The authors declare no conflicts of interest.

## Data Availability

The data that support the findings of this study are available from the corresponding author upon reasonable request.
